# Supplementary material for: TreeCluster: Clustering biological sequences using phylogenetic trees
Source: PLoS One. 2019 Aug 22;14(8):e0221068. doi: 10.1371/journal.pone.0221068 (PMC6705769; doi:10.1371/journal.pone.0221068)
Supplement: S1 Fig — Clustering quality of Greengenes and various TreeCluster modes, where quality is measured as average pairwise distance within a cluster (the lower the better). The horizontal axis shows the number of clusters for a given method and a threshold value. TreeCluster OTUs based on Max-diameter and Sum-length options outperform Single-linkage option as well as Greengenes OTUs. Computation of Hamming distance based cluster diversity for α ≥ 0.7 did not complete within 24 hours and had to be terminated. (PDF) [file pone.0221068.s001.pdf]

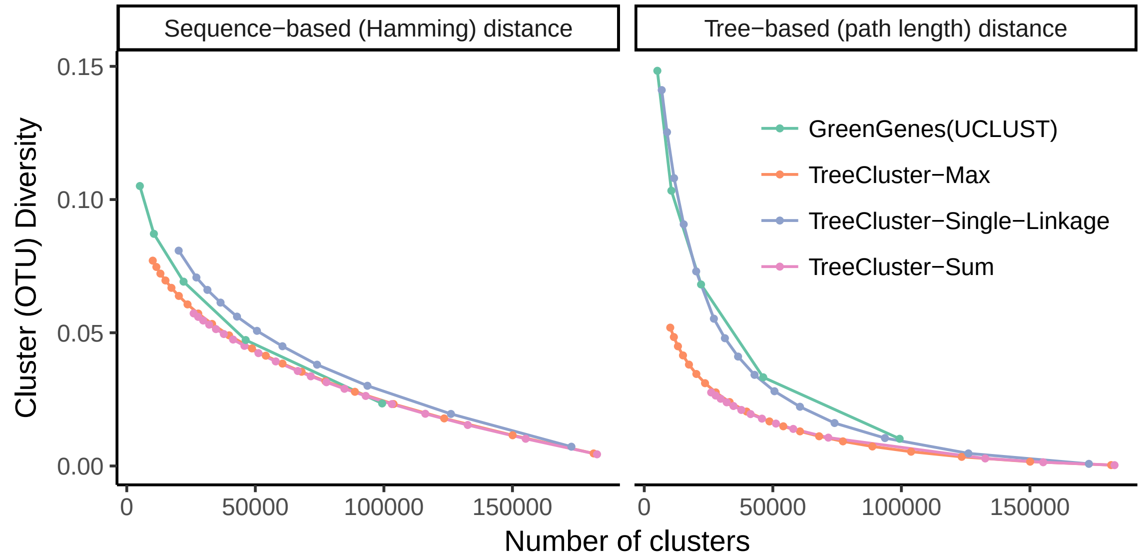

Sequence−based (Hamming) distance
Tree−based (path length) distance
Cluster (OTU) Diversity
0.15
0.10
0.05
0.00
0
50000
100000
150000
Number of clusters
GreenGenes(UCLUST)
TreeCluster−Max
TreeCluster−Single−Linkage
TreeCluster−Sum
