# Supplementary figures and images for: TreeCluster: Clustering biological sequences using phylogenetic trees
Source: PLoS One. 2019 Aug 22;14(8):e0221068. doi: 10.1371/journal.pone.0221068 (PMC6705769; doi:10.1371/journal.pone.0221068)

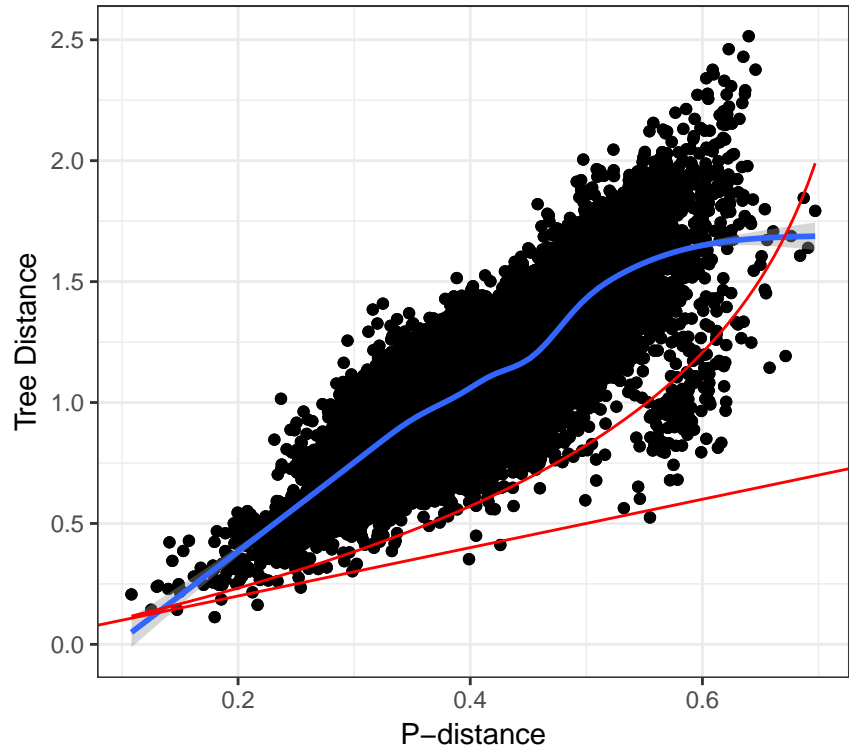

Supplement: S2 Fig — On 16S data, the relationship between tree distances and Hamming distances cannot be established using the Jukes-Cantor formula (red curve). (PDF) [file pone.0221068.s002.pdf]

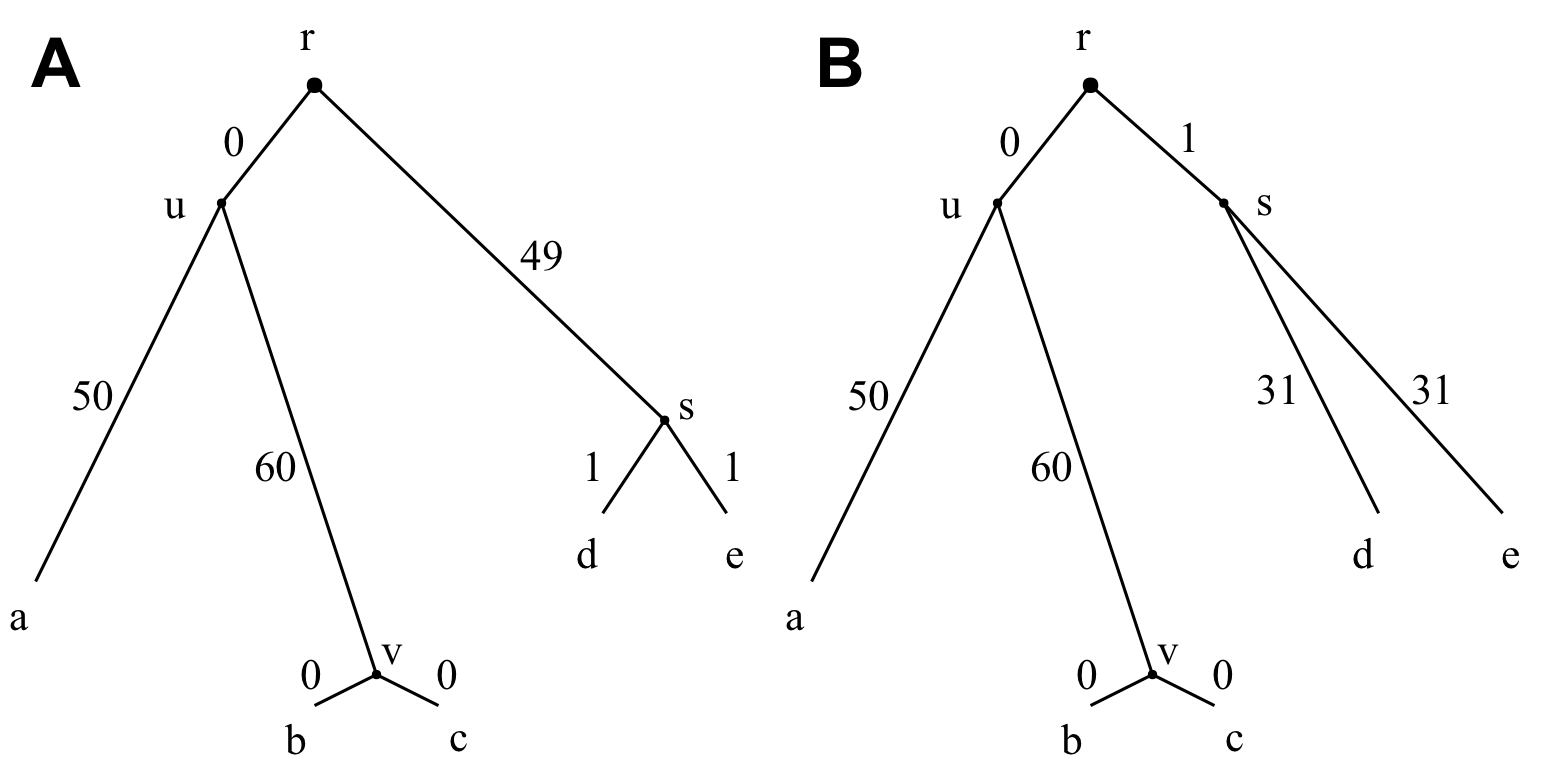

Supplement: S3 Fig — When a greedy algorithm is at the stage where it processes u, it makes the decision for cutting its children edges (u, v) and (u, a) based on the information available at the subtree rooted by u. When α = 72, (A) T1 and (B) T2 require different cut-sets ({(u, v)} and {(u, a)} respectively) for the optimal Mean-diameter partitioning despite the fact that the subtree rooted by u remains unchanged in T1 and T2. (TIFF) [file pone.0221068.s003.tiff]
