# Supplementary material for: TreeCluster: Clustering biological sequences using phylogenetic trees
Source: PLoS One. 2019 Aug 22;14(8):e0221068. doi: 10.1371/journal.pone.0221068 (PMC6705769; doi:10.1371/journal.pone.0221068)
Supplement: S1 Appendix — (PDF) [file pone.0221068.s004.pdf]

# Proofs and supplementary algorithms

## Proofs for the Max-diameter min-cut partitioning problem

*Proof for Theorem 1.* We use induction. The base case for the induction is the simple rooted tree with root $u$ and two leaves $u_l$ and $u_r$. If $w_l + w_r > \alpha$ the algorithm cuts the longer branch whereas if $w_l + w_r \leq \alpha$ no branch is cut. In both cases, the theorem holds.

The inductive hypothesis is that for a node $u$, the algorithm has computed $A(u_l)$, $A(u_r)$, $B(u_l)$, and $B(u_r)$ optimally. We need to prove that a solution other than the one computed by our algorithm *i)* cannot have a lower number of clusters, call it $A'(u)$, and *ii)* when $A'(u) = A(u)$, cannot have a lower distance to the farthest connected leaf, call it $B'(u)$.

When $B(u_l) + w_l + B(u_r) + w_r \leq \alpha$, we have $A(u) = A(u_l) + A(u_r) - 1$, which is the minimum possible by inductive hypothesis and the fact that the number of clusters cannot go down by more than one on node $u$. Also, $B(u)$ is optimal by construction.

When $B(u_l) + w_l + B(u_r) + w_r > \alpha$, without loss of generality, assume that $B(u_l) + w_l \geq B(u_r) + w_r$ and thus, the algorithm cuts the $(u, u_l)$ branch, getting $A(u) = A(u_l) + A(u_r)$ and $B(u) = B(u_r) + w_r$. Note that $A'(u) < A(u)$ is only possible if $A'(u_l) = A(u_l)$ and $A'(u_r) = A(u_r)$ and we do not cut any branch at $u$ in the alternative clustering. However, this scenario is *not* possible because

$$B'(u_l) + w_l + B'(u_r) + w_r \geq B(u_l) + w_l + B(u_r) + w_r > \alpha$$

where the first inequality follows from the inductive hypothesis and the final inequality shows that we will have to cut a branch in any alternative setting. Finally, we need to show that an alternative solution with $A'(u) = A(u)$ but $B'(u) < B(u)$ is not possible. The inequality requires that either $B'(u_l) < B(u_l)$ or $B'(u_r) < B(u_r)$. First, consider the $B'(u_l) < B(u_l)$ case, which is possible only if $A'(u_l) = A(u_l) + 1$. Note that $A'(u) = A(u)$ requires $A'(u_r) = A(u_r)$ (and thus $B'(u_r) = B(u_r)$) and that $B'(u_l) + w_l + B(u_r) + w_r < \alpha$, which is possible. Under this condition, we find:

$$B'(u) = max(B'(u_l) + w_l, B(u_r) + w_r) \geq B(u_r) + w_r = B(u) \quad (4)$$

If instead $B'(u_r) < B(u_r)$, similar conditions can be written, resulting in

$$B'(u) = max(B(u_l) + w_l, B'(u_r) + w_r) \geq B(u_l) + w_l \geq B(u_r) + w_r = B(u) \quad (5)$$

Thus, $A(u)$ and $B(u)$ are optimal when $B(u_l) + w_l + B(u_r) + w_r > \alpha$.

□

*Proof for Corollary 1.* Let $o_r$ and $o_l$ denote the right and the left child of the root of $T^o$. Every edge in $T$ can be mapped to $T^o$ except the edge $(o_r, o_l)$, from which we define a mapping to $(o, o_r)$ (w.l.o.g). Using this mapping, the optimal clustering (i.e., optimal cut-set) on $T$ can be translated to an alternative Max-diameter min-cut partitioning on $T^o$. However, by Theorem 1, $A(o)$ is optimal and cannot be improved by any alternative partitioning. Since any admissible clustering on $T^o$ is also admissible on $T$, Algorithm 1 minimizes the number of clusters.

□

## Linear-time solution for the Sum-length min-cut partitioning problem

We now show that Algorithm A is correct. Let $A(u)$ be the minimum number of clusters under $U$ all with a diameter less than $\alpha$; i.e., $A(o)$ is the objective function.

**Algorithm A:** Linear-time solution for Sum-length min-cut partitioning

**Input:** A tree $T^o = (V, E)$ and a threshold $\alpha$

$B(u) \leftarrow 0$ **for** $v \in V$
**for** $u \in$ *post order traversal of internal nodes of* $T^o$ **do**
  **if** $B(u_l) + w_l + B(u_r) + w_r > \alpha$ **then**
    **if** $B(u_l) + w_l \leq B(u_r) + w_r$ **then**
      $E \leftarrow E - \{(u, u_r)\}$
      $B(u) \leftarrow B(u_l) + w_l$
    **else**
      $E \leftarrow E - \{(u, u_l)\}$
      $B(u) \leftarrow B(u_r) + w_r$
  **else**
    $B(u) \leftarrow B(u_l) + w_l + B(u_r) + w_r$
**return** *Leafsets of every connected component in* $T^o$

**Theorem A.** *Algorithm 1 computes a clustering with minimum $A(o)$ for rooted tree $T^o$. In addition, among all possible such clusterings, the algorithm picks the solution with minimum $B(o)$.*

*Proof.* The proof uses induction. The base case for the induction is the simple rooted tree with root $u$ and two leaves $u_l$ and $u_r$. If $w_l + w_r > \alpha$, the algorithm cuts the longer branch, whereas if $w_l + w_r \leq \alpha$, no branch is cut. In both cases, the theorem holds.

The inductive hypothesis is that, for a node $u$, the algorithm has computed $A(u_l)$, $A(u_r)$, $B(u_l)$, and $B(u_r)$ optimally. We need to prove that a solution other than the one computed by our algorithm *i*) cannot have a lower number of clusters, call it $A'(u)$, and *ii*) when $A'(u) = A(u)$, cannot have a lower distance to the farthest connected leaf, call it $B'(u)$.

When $B(u_l) + w_l + B(u_r) + w_r \leq \alpha$, we have $A(u) = A(u_l) + A(u_r) - 1$, which is the minimum possible by the inductive hypothesis along with the fact that the number of clusters cannot decrease by more than one on node $u$. Also, $B(u)$ is optimal by construction.

When $B(u_l) + w_l + B(u_r) + w_r > \alpha$, without loss of generality, assume that $B(u_l) + w_l \geq B(u_r) + w_r$, and thus, the algorithm cuts the $(u, u_l)$ branch, resulting in $A(u) = A(u_l) + A(u_r)$ and $B(u) = B(u_r) + w_r$. Note that $A'(u) < A(u)$ is only possible if $A'(u_l) = A(u_l)$ and $A'(u_r) = A(u_r)$ and we do not cut any branch at $u$ in the alternative clustering. However, this scenario is *not* possible because

$$B'(u_l) + w_l + B'(u_r) + w_r \geq B(u_l) + w_l + B(u_r) + w_r > \alpha$$

where the first inequality follows from the inductive hypothesis and the final inequality shows that we will have to cut a branch in any alternative setting. Finally, we need to show that an alternative solution with $A'(u) = A(u)$ but $B'(u) < B(u)$ is not possible. The inequality requires that either $B'(u_l) < B(u_l)$ or $B'(u_r) < B(u_r)$. First, consider the $B'(u_l) < B(u_l)$ case, which is possible only if $A'(u_l) = A(u_l) + 1$. Note that $A'(u) = A(u)$ requires $A'(u_r) = A(u_r)$ (and thus $B'(u_r) = B(u_r)$) and that $B'(u_l) + w_l + B(u_r) + w_r < \alpha$, which is possible. Under this condition, we find

$$B'(u) = B'(u_l) + w_l + B(u_r) + w_r \geq B(u_r) + w_r = B(u) \tag{6}$$

If, instead, $B'(u_r) < B(u_r)$, similar conditions can be written, resulting in

$$B'(u) = B(u_l) + w_l + B'(u_r) + w_r \geq B(u_l) + w_l \geq B(u_r) + w_r = B(u) \tag{7}$$

Thus, $A(u)$ and $B(u)$ are optimal when $B(u_l) + w_l + B(u_r) + w_r > \alpha$.

□

**Corollary A.** *Let $C'$ be the cut-set obtained by running Algorithm 1 on any arbitrary rooting $T^o$ of unrooted tree $T$. $C'$ optimally solves the Max-diameter min-cut partitioning problem.*

*Proof.* Let $o_r$ and $o_l$ denote the right and the left child of the root of $T^o$. Every edge in $T$ can be mapped to $T^o$ except the edge $(o_r, o_l)$, from which we define a mapping to $(o, o_r)$ (w.l.o.g). Using this mapping, the optimal clustering (i.e., the optimal cut-set) on $T$ can be translated to an alternative Max-diameter min-cut partitioning on $T^o$. However, by Theorem 1, $A(o)$ is optimal and cannot be improved by any alternative partitioning. Since any admissible clustering on $T^o$ is also admissible on $T$, Algorithm 1 minimizes $q$.

□

## Proofs for the Single-linkage min-cut partitioning problem

*Proof of Proposition 1.* ($\Leftarrow$) If $d(a,b) \le \alpha$ but $a$ and $b$ are in distinct clusters $L_a$, $L_b$ respectively, $N$ can be reduced by one by simply merging $L_a$ and $L_b$. $f_T(L_a \cup L_b) \le \alpha$ is satisfied if for any split of $L_a \cup L_b$, there exists a pair of leaves that are from distinct splits and are within $\alpha$ threshold. For any pair of non-empty sets $S$ and $S'$ that satisfy $S \subset L_a$ and $S' \subset L_b$, we have $\min_{j \in S \cup S', k \in (L_a \cup L_b) - (S \cup S')} d(j,k) \le \min_{j \in S, k \in L_a - S} d(j,k) \le \alpha$ and $\min_{j \in S \cup (L_b - S'), k \in S' \cup (L_a - S)} d(j,k) \le \min_{j \in S, k \in L_a - S} d(j,k) \le \alpha$. On the other hand, $\min_{j \in L_a, k \in L_b - S} d(j,k) \le d(a,b) \le \alpha$. This concludes that for $L = L_a \cup L_b$, $f_T(L) \le \alpha$ is satisfied. $L_a$ and $L_b$ can still be merged if the chain $\mathcal{H}$ described above exists. It is trivial to show that there is a link $\langle c_i, c_{i+1} \rangle$ in $\mathcal{H}$ such that $c_i \in L_a$ and $c_{i+1} \notin L_a$. Using the argument above, we can iterate over $\mathcal{H}$ and keep merging clusters (and decrease N) every time we see such a link until we finally merge $L_a$ with $L_b$.

($\Rightarrow$) We describe a procedure to compute the chain $\mathcal{H}$. If $a$ and $b$ in the same cluster $L$, $\min_{k \in L - \{a\}} d(a,k) \le \max_{S \subset L} \{ \min_{j \in S, k \in L - S} d(j,k) \} \le \alpha$ holds, implying that there is a leaf $c_1$ in set $L - \{a\}$ such that $d(a, c_1) \le \alpha$. If $c_1 = b$, theorem follows. If $c_1 \ne b$, we union $a$ and $c_1$, call the union set $L_a$, and add the link $a \to c_1$ to $\mathcal{H}'$. Iteratively, we find the pair $\langle j, k \rangle$ that yields to $\min_{j \in L_a, k \in L - L_a} d(j,k)$, add the link $j \to k$ to $\mathcal{H}'$, and add $k$ to $L_a$ until we finally add $b$ to $L_a$. The elements forming the path between $a$, and $b$ in $\mathcal{H}'$, which can be computed using depth-first-search, constitute a valid chain $\mathcal{H}$. □

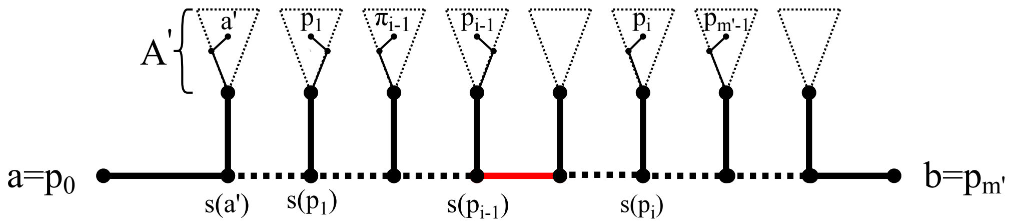


**Fig SA. A sketch showing the setup for constructing the chain $\mathcal{H}$.**

*Proof for Theorem 2.* Let $a \leftrightsquigarrow b$ be the path between leaves $a$ and $b$ on $T$. Fixing $a$ and $b$, for each node $j$, we use the term *support of* $j$, denoted by $s(j)$, to refer to the unique node on all the three paths $a \leftrightsquigarrow b$, $a \leftrightsquigarrow j$, and $b \leftrightsquigarrow j$. We refer to a group of leaves that share a mutual support with respect to $a$ and $b$ as a *bubble* (e.g. triangles in

Fig SA). Among all bubbles branching out of $a \leftrightsquigarrow b$, let the one with the closest support to $a$ be $A'$. We name the leaf closest to $a$ on $A'$ as $a'$ (Fig SA).

We start with the observation that if $d(a,b) \leq \alpha$ holds, the algorithm will never cut any edge on $a \leftrightsquigarrow b$. For every internal node $u$ on $a \leftrightsquigarrow b$, let $v$ and $w$ be the adjacent nodes on $a \leftrightsquigarrow u$ and $u \leftrightsquigarrow b$, respectively. Also, let $p_a$ be the closest leaf to $u$ whose support $s(p_a)$ is on $a \leftrightsquigarrow u$, and let $p_b$ be the closest leaf to $u$ whose support $s(p_b)$ is on $u \leftrightsquigarrow b$. Now, note that $d(p_a,u) + d(u,p_b) \leq d(a,u) + d(u,b) \leq \alpha$ holds, so regardless of the rooting, $(v,u)$ and $(u,w)$ are never cut by Algorithm 2.

If a chain $\mathcal{H}$ exists, due to the previous observation, there are no cuts on $c_i \leftrightsquigarrow c_{i+1}$ for every $0 \leq i \leq m$. Consequently, $a$ and $b$ are connected through a path and are thus in the same cluster.

Assume Algorithm 2 places $a$ and $b$ on the same cluster, i.e., it does not cut any edge on $a \leftrightsquigarrow b$. We present a procedure to generate a chain $\mathcal{H}$ as described in Definition 5. But we first need some definitions. We define $p_0 = a$ and $p_{m'} = b$. For $1 \leq i \leq m'$, let $p_i$ denote the closest leaf to $p_{i-1}$ whose support $s(p_i)$ is on $p_{i-1} \leftrightsquigarrow b$ and $s(p_i) \neq s(p_{i-1})$; i.e., $p_i$ is in the bubble to the right of the bubble of $p_{i-1}$. Conversely, for $1 \leq i \leq m'$, let $\pi_i$ denote the closest leaf to $p_i$ whose support is on $a \leftrightsquigarrow s(p_{i-1})$; i.e., is in a bubble to the left of $p_i$. Also define $\pi_1 = a$. We can also show that every $\pi_i \in \{p_0 \dots p_{i-1}\}$. If a $\pi_i$ is not equal to one of $\{p_0 \dots p_{i-1}\}$, then, $s(\pi_i)$ has to be on $s(p_{j-1}) \leftrightsquigarrow s(p_j)$ for some $j$. However, we would have $d(p_{j-1}, \pi_i) \leq d(p_{j-1}, p_j)$, which contradicts the definition of $p_i$.

Now we construct the chain. The fact that Algorithm 2 retains $(a, s(a'))$ indicates that $min(d(a,a'), d(a,p_1)) = d(a,p_1) \leq \alpha$; therefore, we add $a \rightarrow p_1$ to an auxiliary graph $\mathcal{H}'$. Now, consider Algorithm 2 when it processes the node $s(p_{i-1})$ for $1 < i$. The fact that the first edge on path $s(p_{i-1}) \leftrightsquigarrow s(p_i)$ (shown in red in Fig SA) is not cut indicates that either $d(\pi_{i-1}, p_i) \leq \alpha$ or $d(p_{i-1}, p_i) \leq \alpha$. Depending on which is true, we add a link from $\pi_{i-1} \rightarrow p_i$ or $p_{i-1} \rightarrow p_i$ to $\mathcal{H}'$. We repeat this process for all $i$ until we reach $i = m'$, where we add an edge to $p_{m'} = b$. Noting that $\pi_i \in \{p_0 \dots p_{i-1}\}$, the $\mathcal{H}'$ graph becomes a directed tree, rooted at $a$ with a directed path to the leaf $b$. This directed path constitutes the valid chain $\mathcal{H}$. $\square$

## Mean-diameter clustering with clade constraint

**Algorithm B:** AVERAGE DIAMETER CLADE Average diameter clade min-cut partitioning

**for** $u \in$ *post order traversal of* $T^o$ **do**
- $totPairDist[u] \leftarrow 0$; $totLeafDist[u] \leftarrow 0$;
- **if** $u$ ***in*** $\mathcal{L}$ **then**
  - $numLeaves[u] \leftarrow 1$; $avgPairDist[u] \leftarrow 0$;
- **else**
  - $numLeaves[u] \leftarrow numLeaves[u_l] + numLeaves[u_r]$;
  - $totPairDist[u] \leftarrow totPairDist[u_l] + totPairDist[u_r] + totLeafDist[u_l] \times numLeaves[u_r] + totLeafDist[u_r] \times numLeaves[u_l]$;
  - $totLeafDist[u] \leftarrow totLeafDist[u_l] + w_l \times numLeaves[u_l] + totLeafDist[u_r] + w_r \times numLeaves[u_r]$;
  - $avgPairDist[u] \leftarrow totPairDist[u] / \binom{numLeaves[u]}{2}$;

$toExplore \leftarrow$ queue containing the root of $T^o$;

**while** $toExplore \neq \emptyset$ **do**
- $curr \leftarrow toExplore.dequeue()$;
- **if** $u$ ***not in*** $\mathcal{L}$ ***and*** $avgPairDist[u] > \alpha$ **then**
  - $E \leftarrow E \setminus (u, u_l)$; $E \leftarrow E \setminus (u, u_r)$;
  - $toExplore.enqueue(u_l)$; $toExplore.enqueue(u_r)$;

**return** *Leafsets of every connected component in* $T^o$

## All optimal solutions

**Lemma A.** *Let $\{e_1, e_2, \cdots, e_m\}$ be the set of edges in an unrooted tree $T$. Consider the following algorithm: root $T$ at $e_j$ and run Algorithm 1, and let $\mathcal{S}_j$ denote the set of edges cut by the algorithm in this run. Any optimal clustering for $T$ has to draw its cut-set from $\Sigma = \cup_{j=1}^{m} \mathcal{S}_j$.*

*Proof.* The proof is by contradiction. Assume there is an optimal cut-set $\mathcal{S}'$ that contains an edge $e_i$ such that $e_i \notin \Sigma$. Consider the rooting of $T$ at $e_i$. Denote the root of this tree as $v$, the immediate left and right branches of $v$ as $e_l$ and $e_r$, and the left and right child nodes of $v$ as $v_l$ and $v_r$. Note that the concatenation of $e_l$ and $e_r$ corresponds to $e_i$ in $T$; thus, $e_l \notin \mathcal{S}_j$ and $e_r \notin \mathcal{S}_j$. When $e_i$ is removed from $T$, two new trees form, called $T_l$ (the one containing the node $v_l$) and $T_r$ (the one containing the node $v_r$). If $p$ cuts in $\mathcal{S}'$ are in $T_r$, and if $q$ cuts in $\mathcal{S}'$ are in $T_l$, then $|\mathcal{S}'| = p + q + 1$. The number of cuts in $\mathcal{S}'$ and $\mathcal{S}_j$ are equal, and $e_l$ and $e_r$ are not cut, which implies that either the tree rooted by $v_l$ or $v_r$ has an alternative clustering with one less cut. By the design of Algorithm 1, if this was the case, the algorithm would have chosen the alternative cut. □

# Commands and parameters

**Ancestral state reconstruction using TreeTime.** Each cluster tree is first rooted at its balance point using MinVar rooting version (commit 8c1581a):

```
$ python FastRoot.py -i unrooted_tree.nwk -m MV
-o rooted_tree.nwk
```

Before performing maximum likelihood ancestral state reconstruction, we inferred GTR parameters from the input tree using RAxML v8.2.12:

```
$ raxmlHPC-PTHREADS -f e -t ../input_tree.nwk -s aln.fa
-m GTRGAMMA -n tre -T 4
```

We manually hardcoded those parameters into built-in TN93 parameters matrix in treetime software (v0.5.5) and reconstructed ancestral states of rooted tree using this command:

```
$ treetime ancestral --tree rooted_tree.nwk --aln aln.fa
--outdir outdir --gtr TN93
```

**Listing A.** Default FAVITES Parameters

```
{
    "ContactNetworkGenerator": "Communities",
    "cn_generators": [{
        "ContactNetworkGenerator": "BarabasiAlbert",
        "num_cn_nodes": 5000,
        "num_edges_from_new": 5
    }]*20,
    "cn_p_across": 1/(2*19*5000),
    "NodeEvolution": "VirusTreeSimulator",
    "vts_growthRate": 2.851904,
    "vts_max_attempts": 100,
    "vts_model": "logistic",
    "vts_n0": 1,
    "vts_t50": -2,
    "NumBranchSample": "Single",
    "NumTimeSample": "Once",
    "SeedSelection": "Random",
    "num_seeds": 15000,
    "SeedSequence": "VirusNonHomYuleHeightGTRGamma",
    "seed_height": 25,
    "seed_speciation_rate_func": "exp(-t**2)+1",
    "viral_sequence_type": "HIV1-B-DNA-POL-LITTLE",
    "seqgen_freq_a": 0.392,
    "seqgen_freq_c": 0.165,
    "seqgen_freq_g": 0.212,
    "seqgen_freq_t": 0.232,
    "seqgen_a_to_c": 1.765707,
    "seqgen_a_to_g": 9.587649,
    "seqgen_a_to_t": 0.691915,
    "seqgen_c_to_g": 0.863348,
    "seqgen_c_to_t": 10.282617,
    "seqgen_g_to_t": 1.0,
    "seqgen_gamma_shape": 0.405129,
    "seqgen_num_gamma_rate_categories": "",
    "SequenceEvolution": "GTRGammaSeqGen",
    "Sequencing": "Perfect",
    "SourceSample": "Random",
    "TimeSample": "GranichFirstART",
    "TransmissionTimeSample": "HIVARTGranichGEMF",
    "end_time": 10,
    "hiv_freq_ns": 0,
    "hiv_freq_i3": 0,
```

```
"hiv_freq_i4": 0,
"hiv_freq_a3": 0,
"hiv_freq_a4": 0,
"hiv_freq_d": 0,
"hiv_freq_s": 100000-15000,
"hiv_freq_i1": 50,
"hiv_freq_i2": 5094,
"hiv_freq_a1": 9,
"hiv_freq_a2": 15000-50-5094-9,
"hiv_a1_to_a2": 4.333333333333333,
"hiv_a1_to_d": 0,
"hiv_a1_to_i1": 0.48,
"hiv_a2_to_a3": 0,
"hiv_a2_to_d": 0,
"hiv_a2_to_i2": 0.48,
"hiv_a3_to_a4": 0,
"hiv_a3_to_d": 0,
"hiv_a3_to_i3": 0,
"hiv_a4_to_d": 0,
"hiv_a4_to_i4": 0,
"hiv_i1_to_a1": 1.0,
"hiv_i1_to_d": 0,
"hiv_i1_to_i2": 8.666666666666666,
"hiv_i2_to_a2": 1.0,
"hiv_i2_to_d": 0,
"hiv_i2_to_i3": 0,
"hiv_i3_to_a3": 0,
"hiv_i3_to_d": 0,
"hiv_i3_to_i4": 0,
"hiv_i4_to_a4": 0,
"hiv_i4_to_d": 0,
"hiv_ns_to_d": 0,
"hiv_ns_to_s": 999999,
"hiv_s_to_d": 0,
"hiv_s_to_i1_by_a1": 0.005625,
"hiv_s_to_i1_by_a2": 0,
"hiv_s_to_i1_by_a3": 0,
"hiv_s_to_i1_by_a4": 0,
"hiv_s_to_i1_by_i1": 0.1125,
"hiv_s_to_i1_by_i2": 0.0225,
"hiv_s_to_i1_by_i3": 0,
"hiv_s_to_i1_by_i4": 0,
"hiv_s_to_i1_seed": 0,
"TreeUnit": "TruncatedNormal",
"tree_rate_loc": 0.0008,
"tree_rate_max": float('inf'),
"tree_rate_min": 0,
"tree_rate_scale": 0.0005,
"ContactNetwork": "NetworkX",
"Driver": "Default",
"EndCriteria": "GEMF",
"Logging": "File",
```

```
    "NodeAvailability": "Perfect",
    "TransmissionNodeSample": "GEMF",
    "TreeNode": "Simple",
    "gemf_path": "GEMF",
    "hmmemit_path": "hmmemit",
    "java_path": "java",
    "out_dir": "FAVITES_output",
    "seqgen_path": "seq-gen",
}
```
