# Supplementary material for: TreeCluster: Clustering biological sequences using phylogenetic trees
Source: PLoS One. 2019 Aug 22;14(8):e0221068. doi: 10.1371/journal.pone.0221068 (PMC6705769; doi:10.1371/journal.pone.0221068)
Supplement: Attachment — Submitted filename: plosone revision.pdf [file pone.0221068.s005.pdf]

We thank the reviewers for the positive comments. We have responded to the points in detail below. In addition, we have now added a new analysis where we select a centroid node for each cluster and show that this centroid selection performs better than centroids available in GreenGenes.

----------------------- REVIEW 1 ---------------------
PAPER: 17
TITLE: TreeCluster: Clustering Biological Sequences using Phylogenetic Trees
AUTHORS: Metin Balaban, Niema Moshiri, Uyen Mai and Siavash Mirarab

----------- Overall evaluation -----------
The authors attempt to re-invigorate various tree partitioning approaches in conjuction with sequence similarity data. The approach has been looked at the bioinformatics community before, but only in specific context such as ultrametric trees. To this end, the authors describe a few new algorithms and discuss implementation results with these algorithms. I think the paper is both theoretically and empirically interesting, though its potential future impact in bioinformatics is not completely clear.

Response: We thank the reviewer for the positive comments. By providing three specific applications of the method, we aim to make sure the paper will have an impact in practice. In particular, we hope the method will be used in future for defining OTUs. We are currently working with collaborators on applying this method to a different problem, namely designing libraries for targeted capture sequencing.

----------------------- REVIEW 2 ---------------------
PAPER: 17
TITLE: TreeCluster: Clustering Biological Sequences using Phylogenetic Trees
AUTHORS: Metin Balaban, Niema Moshiri, Uyen Mai and Siavash Mirarab

----------- Overall evaluation -----------
This paper presents a new method (or, rather, family of methods) for converting a phylogeny into a collection of sequence clusters, based on a set of principled criteria (such as average diameter). It is shown that these methods require linear time, and can be modified to respect monophyletic requirements. The methods are then applied to three problems: metagenomics clustering, epidemiological clustering, and fast tree construction.

The paper is well-written and coherently argued. I appreciated the theoretical results, and I can certify that they look accurate though I did not have the patience to check them in full. The method also demonstrates its utility in three separate settings, and this is a clear strength. In addition, the code is publicly available (I did not check its execution but I confirm that it is available on GitHub). There is a substantial amount of comparison to existing methods.

The method demonstrates its effectiveness in practice, including on fairly large datasets, and the reported speed of execution is impressive. An additional strength is that not only external comparisons but also internal ones (i.e. which variant of the method works best) are performed; this is helpful in deciding which variant to use. I only have a few minor suggestions to make:

Response: We thank the reviewer for the positive comments.

1) try to separate out the theorems and proofs to improve the flow - right now, some theorems are proven in the text, others in the appendix, and this is not done systematically

Response: Following the suggestion, we moved all proofs to the appendix. We now include algorithm description, pseudocode, and theorem(s) for Max-diameter and Single-linkage min-cut partitioning problem in the main paper. We only include algorithm description for Sum-branch min-cut partitioning problem.

2) provide a more compelling reason why you believe the mean-diameter min-cut is not solvable in O(n) without clade constraints

Response: Following the suggestion, we withdrew our conjecture that mean-diameter min-cut is not solvable in O(n) without clade constraints. We replaced it with the statement that it cannot be solved by the greedy algorithm similar to Algorithm 1. We acknowledge that a non-greedy algorithm similar to the one that solve Single-linkage variant can potentially solve the mean-diameter variant. However, we argued that mean-diameter variant could be ill-defined without clade constraints for most applications.

.

3) in addition to improving PASTA (the authors' own software), can you try to use this to improve other similar software tools?

Response: Not all methods used for MSA use divide-and-conquer. Our method can only help improve divide-and-conquer methods of MSA, and not all MSA methods. We have now clarified this point in section "Three applications of TreeCluster" under Application 3. We were not able to identify other MSA tools that we have not developed and could be easily updated by us and used divide-and-conquer. Changing software developed by other groups is a highly non-trivial task and requires much effort. We are sure the reviewer can understand these difficulties and we hope our clarification is enough to address this comment.

----------------------- REVIEW 3 ---------------------
PAPER: 17
TITLE: TreeCluster: Clustering Biological Sequences using Phylogenetic Trees
AUTHORS: Metin Balaban, Niema Moshiri, Uyen Mai and Siavash Mirarab

----------- Overall evaluation -----------
This paper develops a general approach for the clustering of sequences using a phylogenetic tree. Given such a tree, the (not-so-new) approach seeks to minimize the number of clusters (basically clades) for a given cutoff on either their tree-based diameter, sum-of-branch-lengths, or maximal *subset separation*. It turns out that a linear-time algorithm was already known (at least for the first two criteria; while the third criterion turns out to perform poorly in practice). The paper makes a strong case in favor of the first two criteria by applying them to three different applications in bioinformatics.

Response: We thank the reviewer for the positive comments.

The results are interesting and should help convince bioinformaticians to consider this type of three-based clustering. But the organization of the paper is not optimal. The first half goes into considerable details on the theoretical properties of the algorithms. But this reads like a *report on failures*: two of the *new* methods turn out to have been known for a long time and the third one does not work well. Why spend so much real estate up front on proofs of essentially known results or properties of a not-so-useful method? I would move most of this to an appendix, and ensure that the reader gets quickly to the true contribution, which seems to lie in the applications.

Response: Following the suggestion, we moved all proofs to the appendix. We now include algorithm description, pseudocode, and theorem(s) for Max-diameter and Single-linkage min-cut partitioning problem in the main paper. We only include algorithm description for Sum-branch min-cut partitioning problem.

Small things:

On line 61, I find the claim that tree-based clustering needs revitalization misleading. After all, in two of the three applications presented, previous methods use a tree to help the clustering. I would phrase the contributions of the current submission more carefully.

Response: We agree that the tree-based approach has been previously used in the epidemiologic application (ClusterPicker). In this context, the value of our approach in that case lies in its efficiency (bringing down the time complexity to O(n), from O(n^3) of Clusterpicker) and number of alternative variants. Following the suggestion, we replaced the aforementioned statement with the following one: "Here, we argue that a fast and efficient tree-based clustering approach can be beneficial to several questions in bioinformatics."

Although the first section of Materials and Methods is entitled TreeCluster, the latter is never referred to in the section. What is it exactly? A software package, a particular implementation of these methods? What does it take as input (sequences, tree)? Does it compute the tree itself? Are there user-defined parameters?

Response: We renamed the first section of Materials and Methods as "Algorithms". We created another subsection called "TreeCluster software" in the same section. In that subsection, we report:

"We implemented linear-time algorithms for min-cut partitioning problem subject to Max-diameter, Sum-branch, Single-linkage, and other clustering criteria, with and without clade constraints in a freely-available cross-platform open source Python 3 tool called TreeCluster. TreeCluster takes a newick tree and a threshold value as input, and returns clusters in a formatted text file. TreeCluster uses treeswift package for fast tree operations and also includes several for non-linear time clustering options."

On line 237, *researches* should be *researchers*.
Response: Typo is fixed.

On line 259, *the use* of *clusters*?
Response: Typo is fixed.

On line 441, I think you mean to say that Single-linkage has substantially *higher* diversity.
Response: Typo is fixed.

In Fig S1, are you sure readers will know what *patristic* means?
Response: Titles are changed to "Sequence-based (Hamming) distance","Tree-based (path length) distance" respectively.

----------------------- REVIEW 4 ---------------------
PAPER: 17
TITLE: TreeCluster: Clustering Biological Sequences using Phylogenetic Trees
AUTHORS: Metin Balaban, Niema Moshiri, Uyen Mai and Siavash Mirarab

----------- Overall evaluation -----------

This is a very nice paper applying some basic algorithms from theoretical computer science to the problem of clustering biological sequences. The results are straightforward and the paper is generally well-written. While I have some doubts whether this tool will be used by the potential audiences it targets, certainly the science is of high value and should be published.

Response: We thank the reviewer for the positive comments. By providing three specific applications of the method, we aim to make sure the paper will have an impact in practice. We are currently working on expanding the areas of application such as designing libraries for targeted capture sequencing.

Minor points:

-"maximizes" should be "maximizing" on line 24

Response: Typo is fixed.

-"linear" is misspelled on line 561

Response: Typo is fixed.
